# Spina Ventosa; Isolated tuberculous osteomyelitis of the little finger: A forgotten differential diagnosis

**DOI:** 10.1016/j.idcr.2021.e01316

**Published:** 2021-10-22

**Authors:** Wael Goravey, Gawahir A. Ali

**Affiliations:** Department of Infectious Diseases, Communicable Diseases Centre, Hamad Medical Corporation, Doha, Qatar

**Keywords:** Tuberculous dactylitis, Spina Ventosa, Osteomyelitis, Extra-pulmonary TB

## Abstract

Isolated tuberculous dactylitis is a rare form of extra-pulmonary tuberculosis that frequently eludes assessment and constitutes diagnostic challenges. This case reminds physicians of keeping tuberculosis (TB) as a differential when dealing with chronic finger ulcers to avoid devastating consequences.

A 29-year-old left-handed male, a manual worker, was referred to our hospital complaining of three-month history of a painless ulcer on the left little finger. There was no history of recent trauma or constitutional symptoms. He was treated with multiple courses of antibacterials for a presumptively infected wound. Examination showed a 2.5 cm non-tender ulcer with an erythematous base on the dorsal aspect of the middle phalanx in the left little finger with no discharging pus. The handgrip was affected but there was a good range of joint movements. Laboratory tests were within normal limits except for a CRP level of 10 mg/L (0–5). An X-ray of the hand showed middle phalanx osteomyelitis in the left little finger with no joint involvement (Fig. 1). The initial impression was an infectious etiology or a tumor of the finger. The punch biopsy of the ulcer showed necrotizing granulomatous inflammation (Fig. 2) whereas bacterial and fungal cultures were negative. However, MTB (GeneXpert MTB/RIF) was positive with a negative rifampin resistance gene. Subsequently, fully sensitive MTB was isolated. A CXR demonstrated no pulmonary involvement and the HIV test was negative. Based on the obtained results, he was started on standard 12 months TB therapy with complete healing of the ulcer, recovery of the handgrip, and significant improvement of the bony changes (Fig. 3). The patient had no recurrence one year into follow-up.

**Fig. 1:**
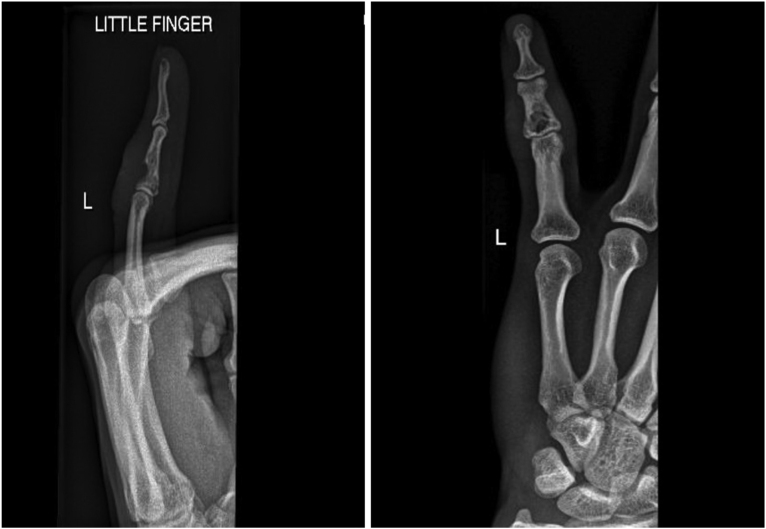
X-rays of the left little finger demonstrating middle phalanx osteomyelitis with no joint involvement.

**Fig. 2:**
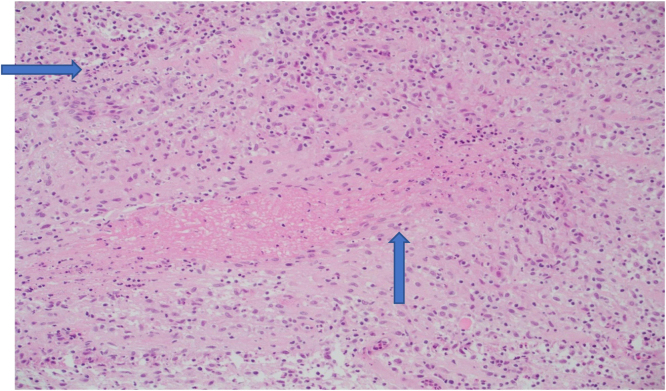
Histopathological examination of the ulcer shows multiple necrotizing granulomatous inflammations, arrowed (H&E ×400).

**Fig. 3:**
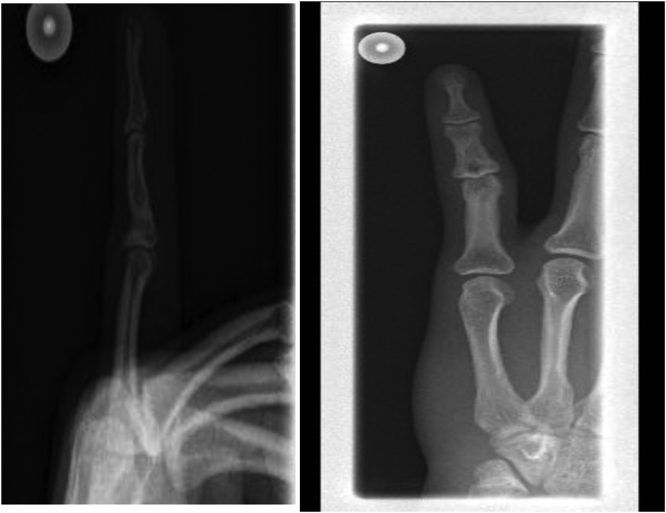
X-rays of the left little finger demonstrating significant interval improvement of the osteomyelitis in the middle phalanx after TB therapy.

Extra-pulmonary TB can affect all sites in the body; hence, isolated TB of the finger bones is not exceptional [1]. Tubercular dactylitis, Spina Ventosa (spina, short bone; Ventosa, inflated with air) represents 2–4% of osteoarticular TB [2]. Children are affected more than adults, bones of the hands are more frequently affected than the feet and the proximal phalanx of the index and middle fingers are the commonest sites for infection [1]. The clinical picture can imitate many infectious and noninfectious conditions leading to morbidity and mortality [3]. A high degree of suspicion with the aid of appropriate tests can clinch the diagnosis and avoid unnecessary consequences. The mainstay of the management is medical and the optimal treatment duration remains unknown but 9–12 months is suggested [1].

## Ethics approval and consent to participate

Ethics approval and permission was obtained to publish the case reports from the institutional review board which is in line with international standards.

## Funding

No funding was received towards the publication.

## CRediT authorship contribution statement

**Gawahir A. Ali:** Clinical management, data acquisition and manuscript writing. **Wael Goravey:** Clinical management, contribute to data acquisition, manuscript preparation and final proof reading.

## Conflict of interest

The authors declare that they have no competing interests.

## Data Availability

The authors confirm that the datasets supporting the findings of this case are available from the corresponding author upon request.
